# Improved chemotherapy modeling with RAG-based immune deficient mice

**DOI:** 10.1371/journal.pone.0225532

**Published:** 2019-11-20

**Authors:** Mark Wunderlich, Nicole Manning, Christina Sexton, Anthony Sabulski, Luke Byerly, Eric O’Brien, John P. Perentesis, Benjamin Mizukawa, James C. Mulloy

**Affiliations:** 1 Division of Experimental Hematology and Cancer Biology, Cancer and Blood Disease Institute, Cincinnati Children’s Hospital Medical Center, Cincinnati, Ohio, United States of America; 2 Division of Hematology and Oncology, Cancer and Blood Disease Institute, Cincinnati Children’s Hospital Medical Center, Cincinnati, Ohio, United States of America; Stanford University, UNITED STATES

## Abstract

We have previously characterized an acute myeloid leukemia (AML) chemotherapy model for SCID-based immune deficient mice (NSG and NSGS), consisting of 5 days of cytarabine (AraC) and 3 days of anthracycline (doxorubicin), to simulate the standard 7+3 chemotherapy regimen many AML patients receive. While this model remains tractable, there are several limitations, presumably due to the constitutional Pkrdc^scid^ (SCID, severe combined immune deficiency) mutation which affects DNA repair in all tissues of the mouse. These include the inability to combine preconditioning with subsequent chemotherapy, the inability to repeat chemotherapy cycles, and the increased sensitivity of the host hematopoietic cells to genotoxic stress. Here we attempt to address these drawbacks through the use of alternative strains with RAG-based immune deficiency (NRG and NRGS). We find that RAG-based mice tolerate a busulfan preconditioning regimen in combination with either AML or 4-drug acute lymphoid leukemia (ALL) chemotherapy, expanding the number of samples that can be studied. RAG-based mice also tolerate multiple cycles of therapy, thereby allowing for more aggressive, realistic modeling. Furthermore, standard AML therapy in RAG mice was 3.8-fold more specific for AML cells, relative to SCID mice, demonstrating an improved therapeutic window for genotoxic agents. We conclude that RAG-based mice should be the new standard for preclinical evaluation of therapeutic strategies involving genotoxic agents.

## Introduction

Currently, NOD/SCID IL2Rγ^-/-^ (NSG) mice are the most commonly used strain for engraftment of both normal and malignant human hematopoietic tissues. These mice represent a dramatic improvement over older strains for engraftment of normal HSCs [1] as well as AML and ALL cell lines and patient samples [2]. NSG mice with transgenic expression of human SCF/GM-CSF/IL-3 cytokines (NSGS) further improved AML engraftment efficiency, latency, and levels [3, 4]. Similarly, NRGS mice (NOD/RAG IL2Rγ^-/-^(NRG) mice harboring the same SCF/GM-CSF/IL-3 transgene) also exhibited improved engraftment of patient AML samples when compared to NRG [5].

We previously characterized the therapeutic response of AML samples to combined Ara-C and doxorubicin in NSGS mice [6]. Importantly, this model revealed differential response of patient samples to a 5+3 regimen; de novo samples showed delayed disease progression while relapse/refractory samples were resistant. This is consistent with the finding of excellent concordance between the response of a large, diverse group of patient derived xenograft (PDX) models to patient outcome using a variety of therapies [7].

While several groups have successfully employed SCID-based immune deficient mice for studies involving PDX response to standard chemotherapies [8–14], there are limitations for doses, frequency, and prior conditioning. These shortcomings are presumably related to the *Prkdc*^*scid*^ mutation, which is responsible for defects in DNA repair [15] and extreme radio-sensitivity [16]. For unknown reasons, these issues are even more pronounced in *IL-2ry*^*-/-*^ mice [1]. In contrast, NRG mice tolerate much higher doses of radiation [17] yet retain the ability to engraft human HSCs and give rise to human blood cell levels and subpopulations that are very similar to NSG mice [18]. It is important to recognize that SCID mutation has functional consequences for every cell in SCID mice, while RAG knockout should only affect differentiation and maturation of lymphocytes. Concerns about SCID-related toxicity are not limited to the hematopoietic compartment for PDX models. For example, it is well established that anthracyclines, which are a common agent in leukemia therapy, have significant toxic effects on cardiac tissues which could be exacerbated in the presence of a SCID mutation [19].

One limitation with previous SCID chemotherapy models was the inability to administer repeated cycles of chemotherapy. Current guidelines for adult and pediatric AML call for two induction cycles, followed by additional intensification/consolidation cycles [20, 21]. Repeated cycles in PDX models may allow for more realistic modeling of response and improved efficacy. Another limitation with the SCID-based model is the inability to give chemotherapy after prior conditioning with either gamma irradiation or busulfan injection. Such conditioning is required for reliably robust engraftment of some PDX samples.

In our previous study, we were careful to examine the effects of chemotherapy on both AML and non-malignant host BM cells [6]. We showed increased sensitivity of AML cells to chemotherapy, particularly with doxorubicin. Ara-C had only minimal selective effects on AML, but increased treatment toxicity. However, these experiments were done in SCID mice, which are likely artificially sensitive to DNA damage-inducing chemotherapy. This sensitivity may artificially lower the relative AML response readout. The maximum tolerable doses of chemotherapies are also likely artificially low and sub-optimal for therapeutic effect.

Recent PDX ALL therapy models in NSG mice utilized a 3-drug induction regimen with vincristine, dexamethasone and L-asparaginase (VXL). This approach has been successfully used along with bioluminescent imaging [22] and combined with Bcl inhibitors [23, 24]. A 4-drug induction protocol (VXL+daunorubicin) optimized for T-ALL engrafted NOD/SCID resulted in 2 of 4 PDX developing signs of resistance [25]. We are unaware of a 4-drug induction protocol for NSG mice. One likely pitfall is increased sensitivity of NSG to anthracyclines [1].

Here, we determined the sensitivity of RAG-based mice (NRG and NRGS) to standard AML and 4-drug ALL induction chemotherapy. RAG-based mice tolerated significantly higher doses of daunorubicin/Ara-C, repeated cycles of therapy as well as combination with busulfan conditioning. Interestingly, we also uncovered a differential activity of doxorubicin and daunorubicin in RAG mice that highlights the importance of full characterization of therapeutics in the various immune deficient models. Finally, we showed that RAG-based host BM cells are more resistant to DA therapy, resulting in an approximate 3.8-fold increase in therapeutic window relative to SCID-based mice. These experiments illustrate the degree to which the choice of host strain may affect results with genotoxic therapies in PDX systems.

## Materials and methods

### Mice

NOD.Cg-Prkdc^scid^Il2rg^tm1Wjl^/SzJ (NSG) and NOD.Cg-Rag1^tm1Mom^Il2rg^tm1Wjl^/SzJ (NRG) mice were obtained from Jackson Laboratories. Generation of NOD.Cg-Prkdc^scid^Il2rg^tm1Wjl^Tg(CMV-IL3,CSF2,KITLG)1Eav/MloySzJ (NSGS) [3] and NOD.Cg-Rag1^tm1Mom^Il2rg^tm1Wjl^Tg(CMV-IL3,CSF2,KITLG)1Eav/J (NRGS) [26] have been previously described. All strains were housed and bred in a pathogen-free facility at Cincinnati Children’s Hospital in accordance with an IACUC protocol. Veternary Services of Cincinnati Children’s Hosptial provided hands on and classroom training concerning proper animal handling for all research staff. Mice (both males and females, aged 8–12 weeks) subjected to chemotherapy protocols were monitored twice daily for signs of toxicity. Mice showing poor mobility, labored breathing, or cumulative weight loss of 30% of their initial body weight were immediately euthanized. These humane endpoints discriminate mice with lethal toxicities from those showing less severe, transient signs of illness from chemotherapy exposure (scruffy appearance and slight hunched posture). Chemotherapy exposed mice were provided moistened food to allow easier feeding and aid hydration. Leukemic mice often rapidly develop hind limb paralysis when tumor burden is high therefore mice with signs of hind limb weakness were also euthanized. Additionally, BM and PB samples were periodically taken from leukemic mice in order to ascertain the level of leukemic burden and to better predict the onset of illness. Bone marrow aspirates were taken from live mice under general anesthesia with isoflurane as previously described in detail [27]. Mice received buprenorphine hydrochloride injections to minimize pain and discomfort before the procedure and after, as necessary. Death was not used as an endpoint for any experiment, however, occasionally mice were found dead, presumably due to rapid progression and onset of disease symptoms and/or toxicities during the overnight hours. This was limited to fewer than 5% of mice involved in our studies. All leukemic and chemotherapy protocols and humane endpoints were reviewed and approved by the Cincinnati Children’s Hospital IACUC prior to study initiation.

### Cells

The MA9.3RAS cell line was generated by sequential retroviral expression of MLL/AF9 and NRasG12D cDNAs into umbilical cord blood (UCB) CD34+ cells, as described previously [3, 28], and was maintained in IMDM/20%FBS. 2X10^5^ cells (i.v. injection) were used to engraft mice for experiments. Upon sacrifice due to AML, control spleen preparations were frozen for later use in the experiments designed to determine AML/BM toxicity. 8-9X10^5^ cells were i.v. injected into non-conditioned mice for these experiments. The AE46T cell line was originally established by sequential retroviral transduction of UCB CD34+ cells with cDNAs encoding AML/ETO and hTERT [29, 30] and was maintained in IMDM/20%FBS supplemented with 10ng/mL SCF, TPO, FLT3-L, IL-3, and IL-6. 1X10^6^ cultured cells were injected i.v. to induce AML.

Patient samples were obtained from patients at Cincinnati Children’s Hospital Medical Center following informed written consent of parents/guardians and assent of patients over 11 years old. Residual diagnostic specimens were used according to a study protocol (#2008–0021) approved by the Cincinnati Children’s Hospital Institutional Review Board (Office for Research Compliance and Regulatory Affairs). Additionally, we used a pre-existing PDX model (frozen spleen from secondary engrafted mice) which was previously generated from cells from a deidentified sample (DFAM-64519-V2, PRoXe.org [31]). Initial primary specimens were incubated with OKT3 antibody to eliminate the potential for xenogeneic GVHD [26]. Following successful engraftment, BM and spleen preparations from primary mice were viably frozen for future experiments. 1–3 X10^6^ thawed cells were injected i.v. for the PDX experiments described in this study. All cell transplants in this study were done by i.v. injection. A table with patient sample and PDX model information is included as supplementary material (S1 Table).

### Chemotherapy

A single i.p. dose of 30mg/kg busulfan was used as a preconditioning regimen in some experiments as described previously [26, 32]. For AML therapy, mice received 1.2mg/kg daunorubicin (D) and 50mg/kg cytarabine (A, Ara-C) by i.v. injection for 3 consecutive days beginning 2–3 weeks after busulfan conditioning and/or cell engraftment (low dose DA therapy). DA was repeated for some mice. Alternatively, a higher dose of 3.0mg/kg daunorubicin and 75mg/kg Ara-C was used (high dose DA therapy). For some experiments, doxorubicin was substituted for daunorubicin. For B-ALL 4-drug induction therapy, we used a 4-week schedule of vincristine (V, 0.5mg/kg, i.p., each Monday), dexamethasone (X, 15mg/kg, i.p., each day Monday-Friday), pegaspargase (P, 1200kU/kg, i.p., 1^st^ and 3^rd^ Monday), and daunorubicin (D, 2.5mg/kg, i.v., each Monday). This treatment is abbreviated “VXPD”. When optimizing chemotherapy doses, mice were monitored for at least 6 weeks after exposure in an attempt to detect longer-term toxicities.

### PB and BM analysis

Tail bleeds were analyzed on a Hemavet9500 (Drew Scientific). Engraftment was determined from flow cytometry of PB and BM preparations using a FACSCantoII instrument (BD) with analysis by FlowJo software. Our standard flow panel consists of antibodies to block mouse and human Fc IgG receptors (Miltenyi Biotech) as well as mCD45-APC/Cy7(BD), CD45-FITC (BD), CD3-PE/Cy7 (BD), CD19-VioBlue (Miltenyi Biotech), CD13-PE (BD), CD33-PE (BD), CD34-APC (BD), and CD56-v510 (BD). Leukemia percentage was determined by calculating the number of cells with positive staining for CD33 and CD45 (AML) or CD19 and CD45 (ALL) as a fraction of viable cells.

### Statistics

Statistics were calculated with Prism 7 software. The Mann-Whitney U test was used to compare 2 groups. 2-way ANOVA was used to compare groups with repeated measurements. Log-rank analysis was used to compare survival curves. Linear regression analysis was performed to compare trendlines.

## Results

### RAG mice tolerate higher doses of AML chemotherapy

**Fig 1:**
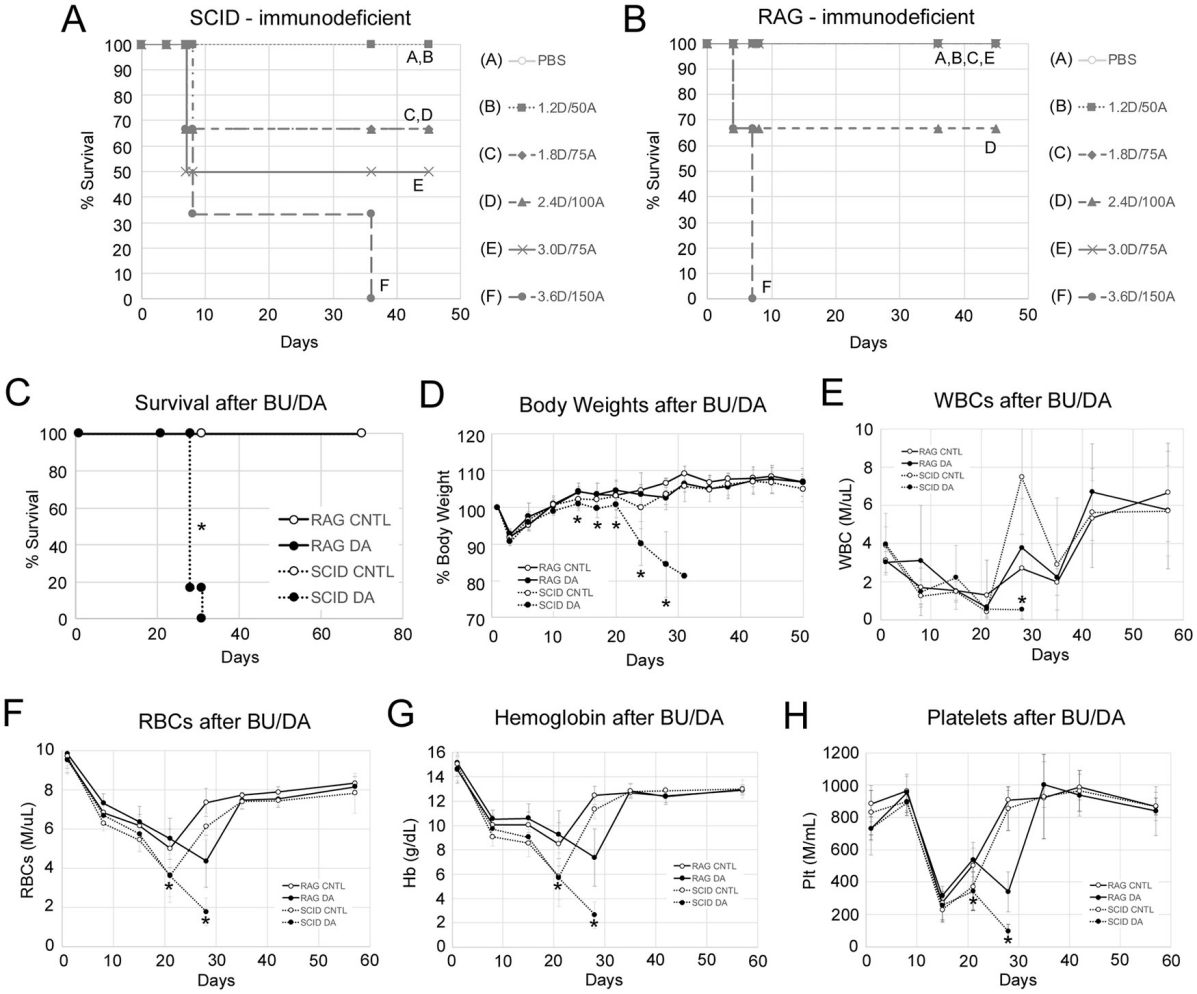
RAG knockout mice tolerate higher AML therapy than SCID-based mice. A) Naïve male NSGS mice (n = 3–4, 25.6 +/- 1.1g average body weight) with SCID immune deficiency or B) NRGS male mice (n = 3–4, 27.8 +/- 2.3g) with RAG knockout immune deficiency were challenged with various doses (doses in mg/kg) of combined daunorubicin (D) and Ara-C (A) injection for 3 consecutive days. Survival was monitored to determine maximum tolerable doses. Mice were sacrificed when they reached humane endpoints as described in the methods. (C-H) Mice (n = 4–5 per group) were conditioned with busulfan 3 weeks before exposure to 1.2mg/kg daunorubicin and 50mg/kg Ara-C (BU/DA). Survival (C), relative body weight (D), PB WBCs (E), RBCs (F), hemoglobin (G), and platelets (H) were monitored for responses to chemotherapy. For C-H, 8–12 week old female mice were used with starting weights of 25.4 +/- 3.0g (NRGS) and 24.4 +/- 1.9g (NSGS). Asterisks indicate p<0.05. For C-H, asterisks indicate significant differences between the SCID DA and RAG DA groups. CNTL = PBS controls, DA = combined daunorubicin/Ara-C, WBCs = white blood cells, RBCs = red blood cells.

We began our comparison of SCID and RAG-based mice by searching for the maximum tolerated dose for combined daunorubicin and Ara-C intravenous infusions over three consecutive days. Our initial chemotherapy model utilized 5 days of Ara-C, however, we found that Ara-C alone produced very little leukemia cell specific killing benefit while adding measurable normal BM toxicity [6]. Therefore, we eliminated the final 2 days of Ara-C exposure. We also switched the anthracycline, replacing doxorubicin with daunorubicin in order to better mimic pediatric AML therapy protocols. NSGS (SCID) mice experienced lethal toxicities at all doses higher than 1.2 mg/kg daunorubicin and 50mg/kg Ara-C (Fig 1A) in line with our previous findings. NRGS (RAG) mice survived a 50% higher dose but succumbed to a double dose of 2.4mg/kg daunorubicin and 100mg/kg Ara-C (Fig 1B). NRGS mice were resistant to increasing daunorubicin to 3.0mg/kg if Ara-C remained at 75mg/kg, implying that Ara-C may contribute more substantially to off-target toxicities. These results establish that RAG mice tolerate substantially higher chemotherapy doses.

Next, we exposed NSGS and NRGS mice to sub-lethal busulfan doses 3 weeks prior to chemotherapy to mimic an approach requiring preconditioning for successful engraftment of leukemia. Preconditioning is required for reliable engraftment of many samples and can significantly speed up disease latency for most. SCID and RAG strains received 1.2mg/kg daunorubicin and 50mg/kg Ara-C for 3 consecutive days. Previously we showed that NSGS mice cannot tolerate a similar 5+3 doxorubicin/Ara-C protocol after either irradiation or busulfan conditioning [6]. Consistent with those findings, the NSGS busulfan+DA group experienced lethal toxicities several days after chemotherapy while similarly-treated NRGS mice survived for the duration of the 5-week post chemotherapy observation period (Fig 1C). The NSGS busulfan+DA group experienced more profound weight loss and failure to recover WBC, RBC, and PLT counts while these parameters returned to baseline levels in NRGS (Fig 1D–1H).

### Optimization of DA therapy in NRGS PDX mice

**Fig 2:**
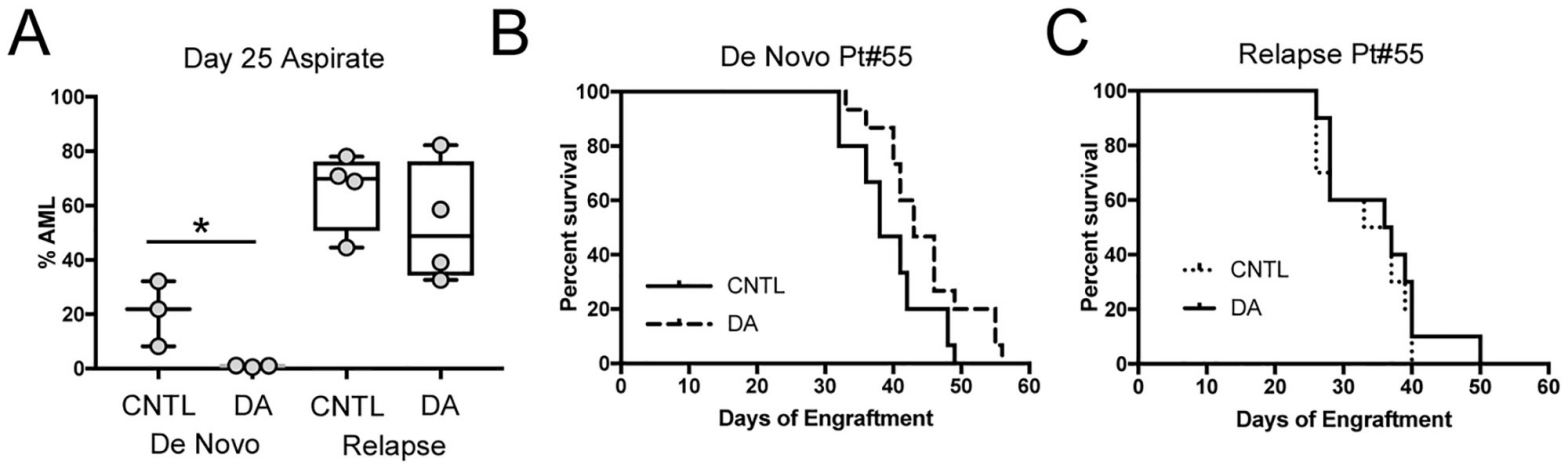
Lack of efficacy with DA in AML-engrafted NRGS. A) NRGS mice were engrafted with PDX samples generated from a paired de novo/relapse AML case. Busulfan was used to pre-condition mice for de novo engraftment, but not for mice receiving the relapse sample. Mice were treated with 1.2mg/mL daunorubicin and 50mg/kg Ara-C at 3 weeks and BM aspirates were analyzed at day 25. Survival of the mice engrafted with the B) de novo and C) relapse PDX samples was monitored. Asterisk indicates p<0.05 by Mann-Whitney U test. CNTL = PBS controls, DA = combined daunorubicin/Ara-C. Mice were randomly assigned to treatment or control groups.

Next, we sought to test the efficacy of combined daunorubicin and AraC in leukemic NRGS mice, with and without prior busulfan conditioning. For this, we used a paired set of de novo and relapse PDX samples from the same patient. Busulfan conditioning was used to aid engraftment of the de novo sample but not the relapse sample. The lower dose of 1.2mg/kg daunorubicin and 50mg/kg AraC (Low Dose, LD) was used because that was the dose successfully tested with busulfan conditioning in Fig 1. Marrow aspirates taken after therapy showed significantly decreased AML levels in the mice harboring the de novo sample, but not in those engrafted with the relapse sample (Fig 2A). However, this effect did not translate into increased survival in the de novo group (Fig 2B). Similarly, DA treatment did not affect survival of the mice with the relapse sample either (Fig 2C).

One possibility for the disconnect between initial treatment response and survival time is that the treatment damages both normal and leukemic cells which then compete to repopulate the bone marrow. If the AML is not sufficiently repressed, then the remaining cells may expand rapidly after therapy and effectively eliminate the gap in AML burden between the treated and control cohorts. Another possibility is that daunorubicin is not as effective as doxorubicin in PDX models. We tested both anthracyclines in two separate approaches to address this lack of efficacy.

**Fig 3:**
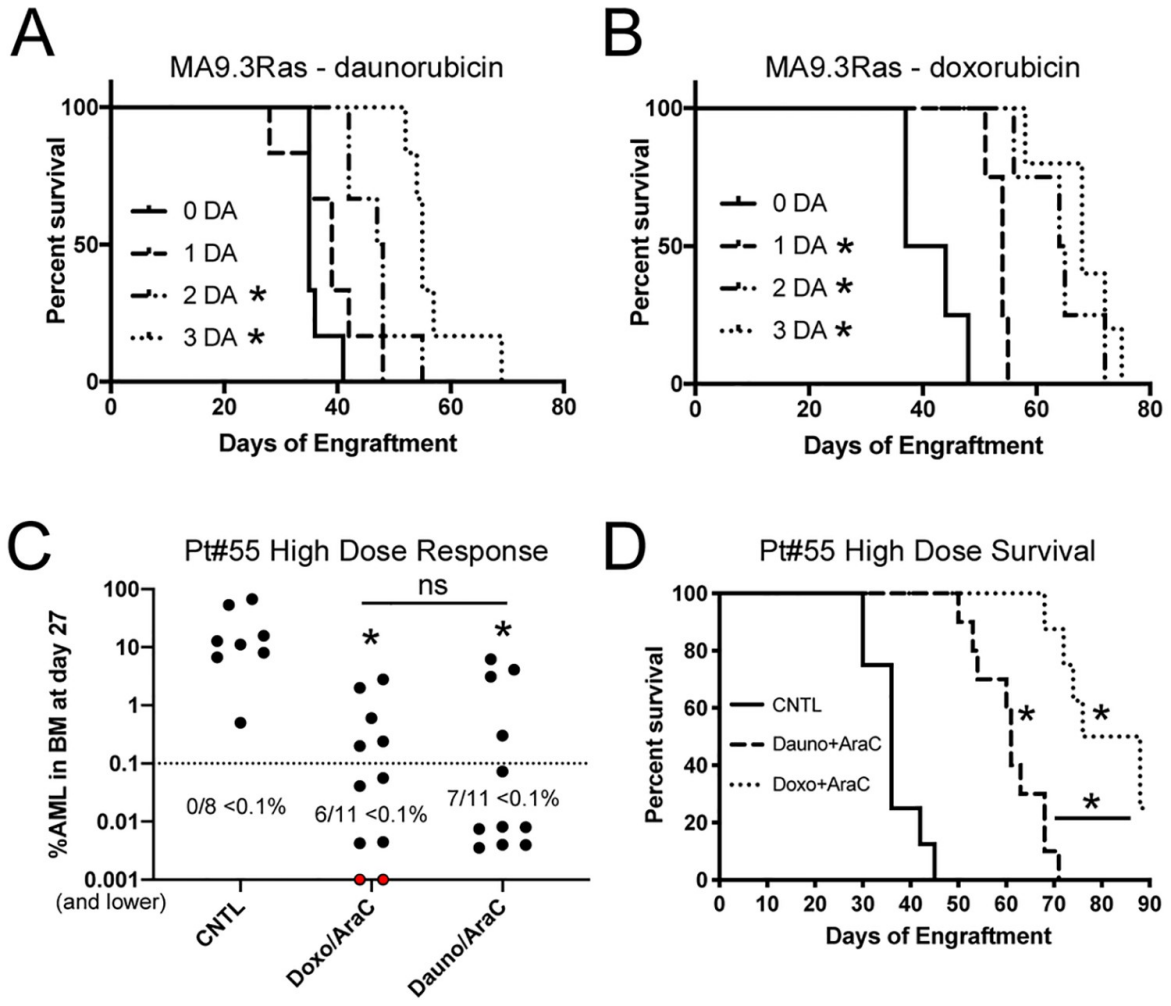
Comparison of doxorubicin to daunorubicin in AML PDX models. A) Survival of NRGS mice engrafted with the MA9.3Ras cell line and treated with 0, 1, 2, or 3 cycles of 1.2mg/kg daunorubicin and 50mg/kg Ara-C (0 DA, 1 DA, 2 DA, 3 DA) or B) 1.5mg/kg doxorubicin and 50mg/kg Ara-C. C) A de novo PDX was engrafted into NRGS mice and treatment began 3 weeks after busulfan conditioning and engraftment using HD DA using either 3.0mg/kg doxorubicin or daunorubicin. AML burden was determined from BM aspirates at day 27. The red points indicate mice with undetectable disease and are plotted as 0.001 in order to include them in the log based plot. D) The mice in C were followed for survival. Log rank tests were used for A,B,D. Mann-Whitney U tests were used to determine significance for C. Asterisks indicate p<0.05. CNTL = PBS controls. Mice in A-D were randomly assigned to treatment or control groups.

First, we tested whether multiple cycles of LD chemotherapy would be tolerated in NRGS mice and improve survival. We engrafted NRGS mice with MA9.3Ras cells and initiated chemotherapy at day 10. After a 1-week break, some mice received a second round of chemotherapy. Others went on to receive a third round according to the same schedule. This schedule of repeated cycles more closely resembles typical patient therapy, which calls for additional therapy in MRD+ or high-risk cases. NRGS mice tolerated additional chemotherapy cycles and survived longer with each successive round of therapy. Consistent with our previous results, a single cycle of 1.2mg/kg daunorubicin and 50mg/kg Ara-C did not show efficacy (Fig 3A). However, when doxorubicin was substituted for daunorubicin, a statistically significant extension of latency was observed with a single cycle which was also further improved by additional cycles (Fig 3B).

Secondly, since we found that RAG mice could tolerate higher chemotherapy doses, we treated NRGS mice engrafted with a chemo naïve PDX sample with 3.0mg/kg daunorubicin or doxorubicin and 75mg/kg AraC (High Dose, HD). NRGS mice tolerated this higher chemotherapy dose 21 days after busulfan conditioning. Mice treated with either HD daunorubicin or doxorubicin (at the same dose) exhibited similar AML burden after therapy (Fig 3C). Approximately half of the mice in each group had AML at less than 0.1% by flow, a clinical cut-off for MRD status. However, most mice did relapse, although survival time was significantly extended (Fig 3D). Notably, doxorubicin resulted in a greater extension of lifespan compared to daunorubicin. In fact, 2 of the 11 HD doxorubicin treated mice had no detectable disease at the end of the experiment.

### Use of HD daunorubicin/AraC in de novo and relapse PDXs

**Fig 4:**
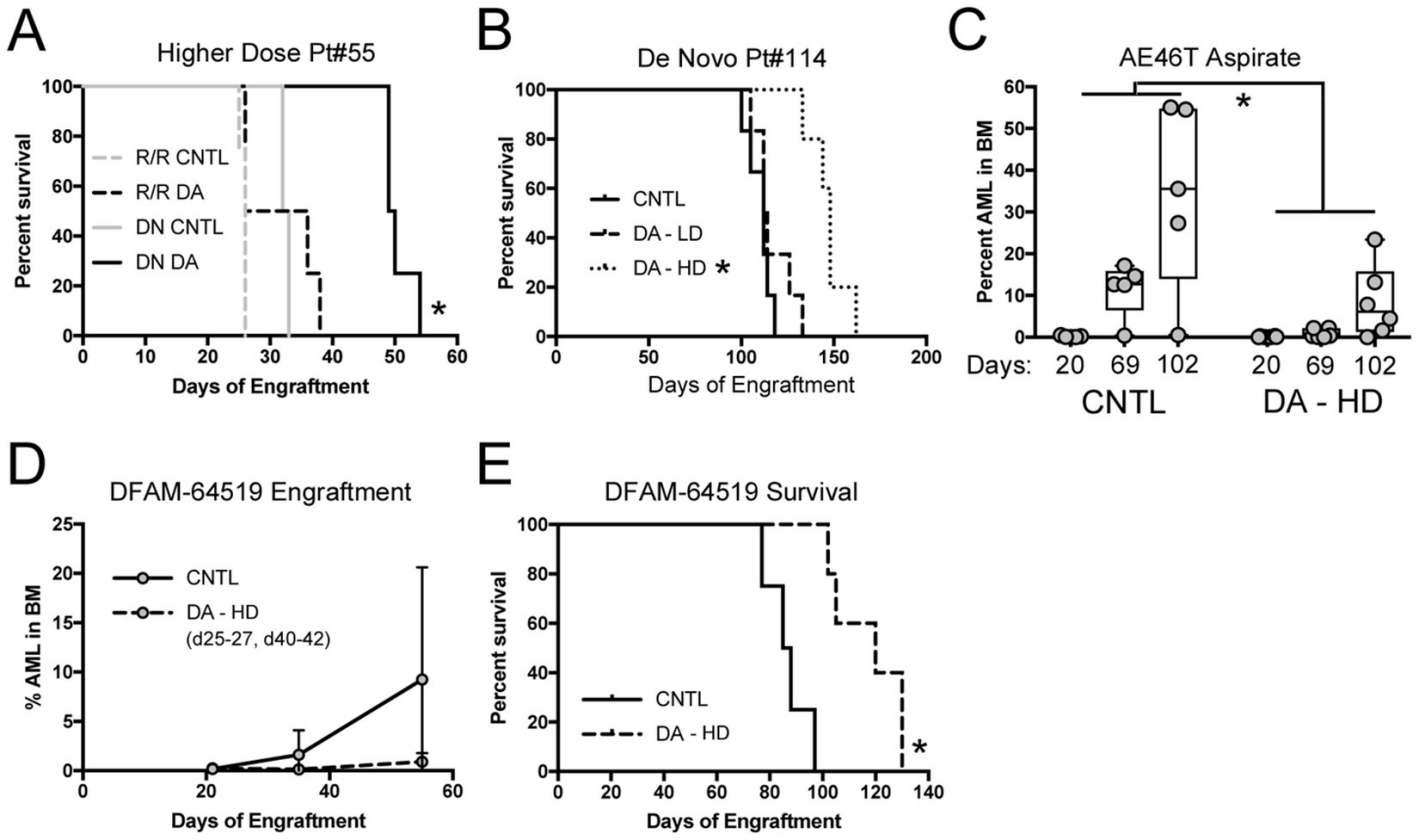
HD chemotherapy for de novo and relapse AML PDXs. A) Survival of NRGS mice engrafted with a matched de novo (DN)–relapsed/refractory (R/R) patient sample were treated with the higher dose of 3.0mg/kg daunorubicin and 75 mg/kg Ara-C. B) Survival of NRGS mice engrafted with a PDX sample from a second de novo case and treated with 1.2mg/kg daunorubicin and 50mg/kg AraC (DA-LD) or a higher dose (DA-HD) as in A. C) NRGS mice engrafted with the AE46T cell line were monitored for AML response to HD DA treatment. D) A relapse adult sample was subjected to two rounds of HD DA chemotherapy. BM AML burden and E) survival are shown. Asterisks indicate p<0.05 by log rank test (panel A, B, E), or 2-way ANOVA (panel C, D). Comparisons are treated versus controls. CNTL = PBS controls. Mice were randomly assigned to treatment or control groups.

We tested the optimized HD chemotherapy treatment protocol in our paired de novo / relapse PDX set. Mice engrafted with the de novo sample responded to therapy with a significantly longer latency while the relapse-engrafted mice showed no response to therapy (Fig 4A). In addition, the HD chemotherapy but not the LD protocol extended the lifespan of busulfan conditioned NRGS mice engrafted with a second chemotherapy-naïve sample (Fig 4B).

We also tested the HD chemotherapy response of mice engrafted with the chemotherapy-naïve AE46T cell line which was derived from UCB CD34+ cells with retroviral directed expression of *RUNX1/RUNX1T1* (*AML1/ETO*) and *TERT* [29]. HD chemotherapy was initiated at day 46, after engraftment was confirmed in the busulfan preconditioned recipients, resulting in delayed progression of leukemia (Fig 4C). We attempted to further delay leukemia by re-treatment at day 110, however the treated mice experienced significant toxicities and the experiment was ended. We repeated this approach with conditioned mice engrafted with a refractory adult MDS/AML sample with the first round of therapy at day 25 followed more closely by a second round of HD DA 2 weeks later. This timing resulted in mice with low tumor burden after therapy and increased lifespan (Fig 4D and 4E). Importantly, treated mice had similar AML in the BM at sacrifice as controls (CNTL, 64.0 +/- 5.9% vs DA, 72.0 +/- 12.1%), suggesting these mice succumbed to leukemia rather than treatment-related toxicities. Together, these results suggest that treatment toxicities increase in severity as tumor burden increases in PDX models.

### RAG mice better tolerate an ALL 4-drug induction protocol

**Fig 5:**
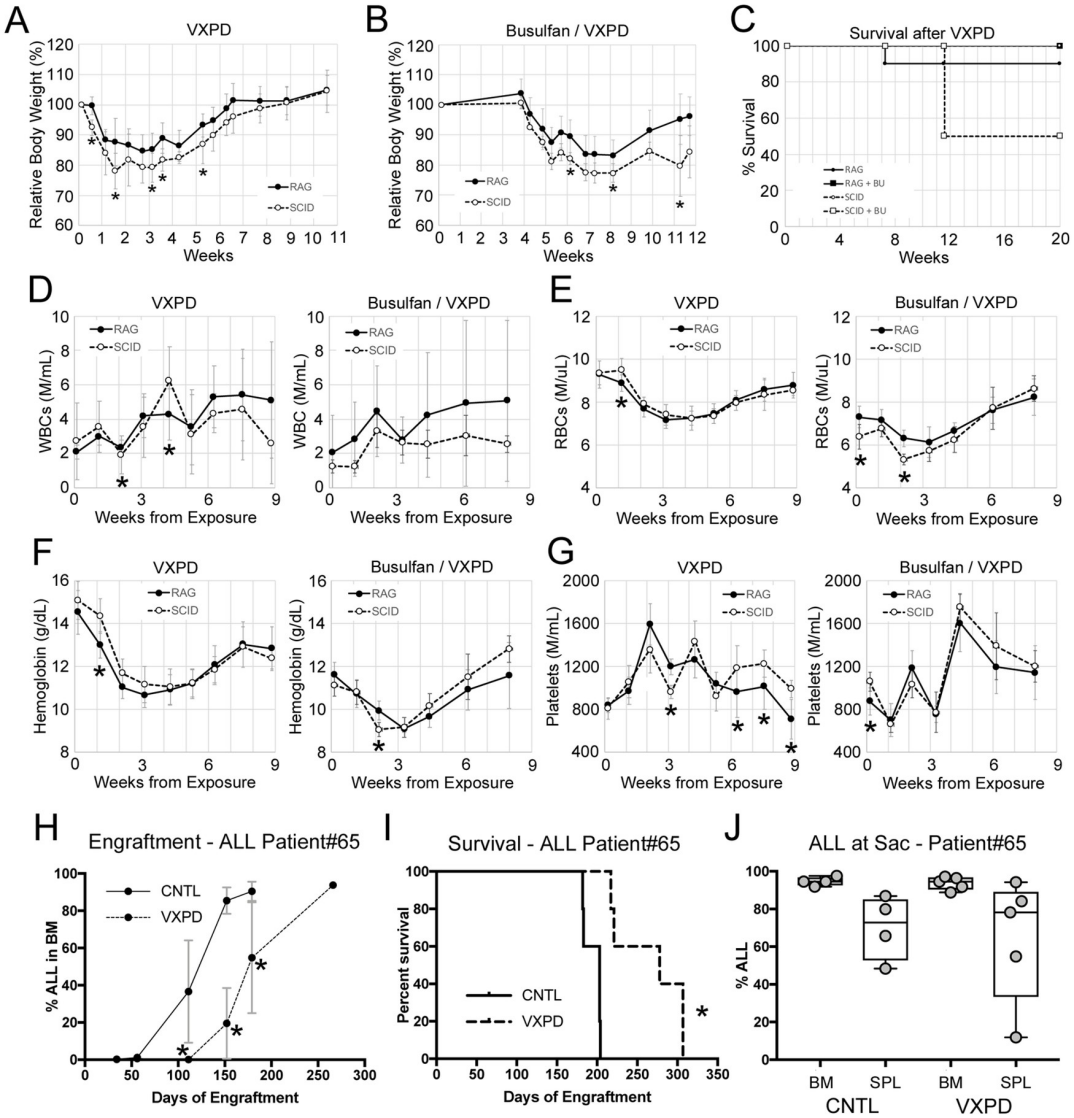
Modeling 4-drug induction for high risk B-ALL. A) Weights of NRG (RAG, n = 10) and NSG (SCID, n = 6) mice were treated with a 4-week course of VXPD. B) Weights of mice conditioned with busulfan 3 weeks prior to VXPD (n = 6 per group). C) Survival of mice in A and B. Mice were sacrificed when they reached humane endpoints as described in the methods. D) WBCs, E) RBCs F) hemoglobin, and G) platelets were monitored before, during, and after VXPD. H) PDX samples from a pediatric B-ALL sample were engrafted into NRG mice after busulfan conditioning. BM aspirates were analyzed by flow cytometry to monitor engraftment. I) Survival and J) B-ALL levels at time of sacrifice were determined for these mice. Asterisks indicate p<0.05 by Mann-Whitney U test (panels A-B, D-H, J) or log rank (panel I). CNTL = PBS controls.

To explore the suitability of RAG-based mice for B-ALL modeling, we examined the durability of NSG and NRG mice to a 4-drug induction protocol for high risk B-lymphoid leukemia. To test for tolerance, we initially exposed non-conditioned, non-leukemic RAG and SCID based mice to vincristine, dexamethasone, pegaspargase, and daunorubicin. SCID-based NSG mice experienced a more dramatic weight loss relative to RAG-based NRG mice, but both strains recovered from the 4-week treatment (Fig 5A). However, when busulfan conditioning was included 3 weeks prior to chemotherapy, half of the SCID-based mice experienced lethal toxicities several weeks post exposure (Fig 5B and 5C). There were no obvious or consistent statistically significant alterations in hematopoietic parameters as measured by CBC analysis, indicating that this effect was unlikely to be related to excessive BM damage or failure and points to non-hematopoietic toxicity (Fig 5D–5G).

To test the efficacy of 4-drug ALL induction, a chemotherapy-naïve B-ALL was engrafted into busulfan conditioned NRG mice. The 4-week treatment started once B-ALL was detectable in the PB. Serial BM aspirations revealed a dramatic decrease in ALL burden in treated mice relative to controls (Fig 5H) which resulted in a significant latency shift (Fig 5I). Importantly, treated mice showed the same level of ALL as control mice at the time of sacrifice, indicating that the mice did not experience treatment-related toxicities (Fig 5J). These data demonstrate the utility of NRG mice in the modeling of high risk 4-drug ALL induction therapy.

### RAG mice offer a better therapeutic window for genotoxic agents

**Fig 6:**
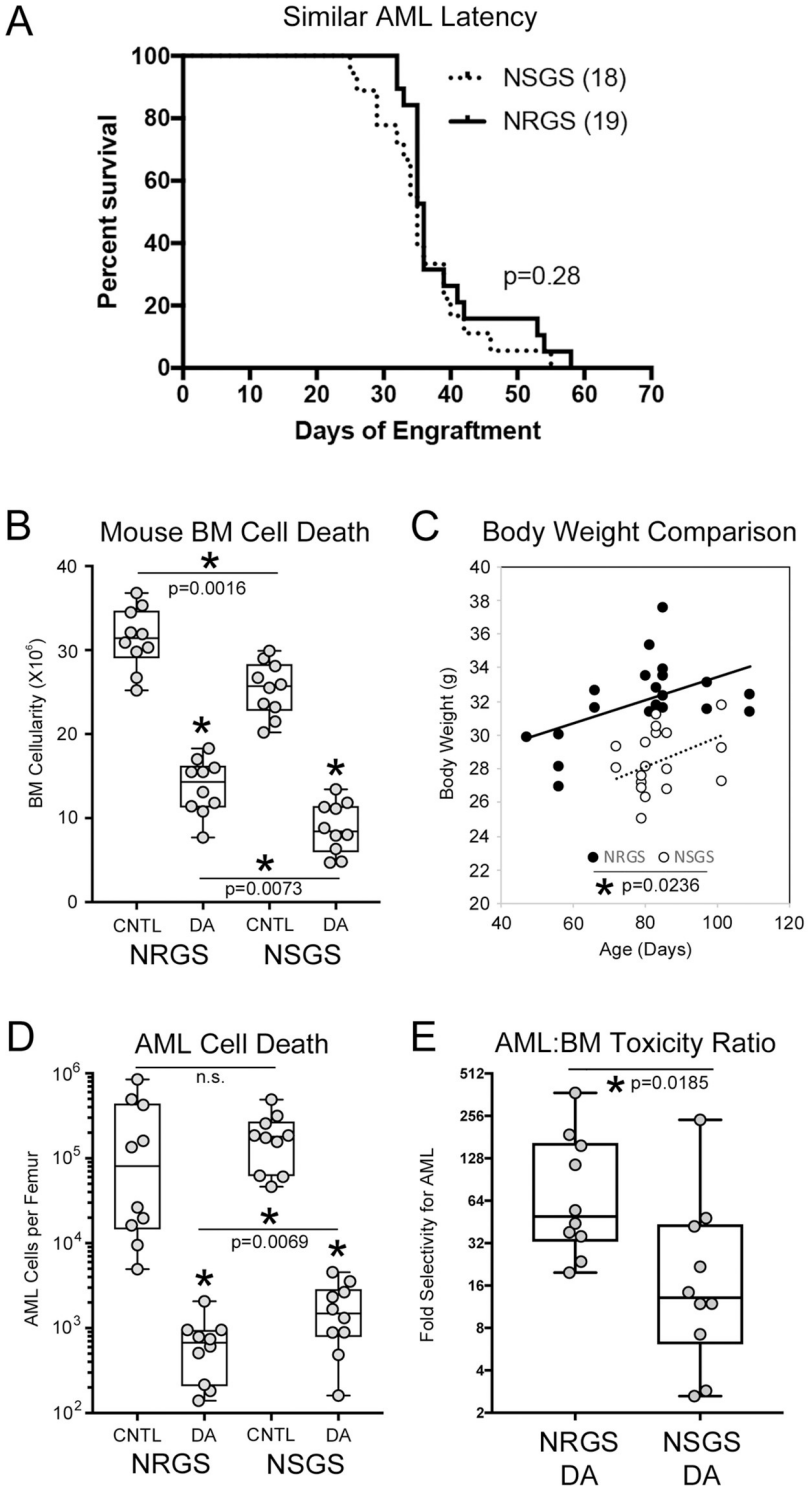
Determination of therapeutic window in NSGS and NRGS mice. A) Latency of MA9.3RAS leukemia induction in NSGS and NRGS mice. B) Day 14 BM cellularity of mice engrafted with MA9.3RAS cells. Mice were treated with 3 days of PBS (CNTL) or DA (1.2mg/kg daunorubicin and 50mg/kg Ara-C) beginning on day 9. Mice were randomly assigned to treatment or control groups. C) Body Weight plotted against age of mice used in panel B. D) Absolute MA9.3RAS cell number in mice presented in panel B. E) The toxicity ratio was calculated for each DA treated mouse. Log rank test was used for panel A. Asterisks indicate p<0.05 by Mann-Whitney U test (panel B, D, and E) or linear regression analysis (panel C).

To this point, we have established that SCID mice have lower tolerance for chemotherapy regimens. We assume that this is at least partly due to the *Prkdc*^*SCID*^ mutation being consequential in all cells. DNA repair should be compromised in SCID mice and therefore we would expect higher rates of apoptosis in response to DNA damaging agents. This problem should be avoided in RAG mice, because RAG knockout should specifically affect lymphocyte development and play no direct role in DNA damage response. The MA9.3Ras cell line causes fatal AML in both NSGS and NRGS with very similar kinetics (Fig 6A), making this model suitable for testing this hypothesis, and for quantifing any SCID/RAG differences. To examine initial response to chemotherapy, we subjected non-conditioned mice engrafted with MA9.3Ras cells to 3 consecutive days of DA exposure and sacrificed them 3 days later. For this experiment we used the SCID MTD of 1.2mg.kg daunorubicin and 50mg/kg AraC (LD). In NSGS DA treated mice, BM cellularity was reduced to 34.7% of controls (25.4 X10^6^ vs 8.8 X10^6^ WBCs/femur) while a somewhat smaller decrease was observed in NRGS (31.4 X10^6^ to 13.7 X10^6^ cells, 43.7%) (Fig 6B). NRGS DA mice had significantly more surviving BM cells than NSGS DA mice (p = 0.0073), however, increased NRGS BM cellularity was also noted in control mice. This finding might be at least partially explained by the overall larger size of age-matched male NRGS compared to NSGS mice (32.0+/-2.3g vs 28.5+/-2.0g, p = 8E-06, Fig 6C). Linear regression analysis of age/weight data confirmed that the NRGS mice used in these experiments were larger than their SCID-based counterparts. Separate analysis of NRG and NSG showed similar significant differences in both age-matched males and females (not shown). Absolute AML cells per femur was decreased in both strains in response to chemotherapy. However, this decrease was more dramatic in NRGS mice, where DA-treated mice contained on average only 0.34%+/-0.26% of control levels while NSGS DA-treated mice retained 0.96%+/-0.73% (p = 0.027, Fig 6D). By taking the percent decrease of normal mouse BM and human AML cell numbers in response to DA together, we calculated the relative AML specific toxicity in both strains. For NRGS mice, DA treatment resulted in a 49.5-fold decline in absolute AML number compared to normal mouse BM cells while NSGS mice experienced only a 13.1-fold difference, suggesting a larger therapeutic window (a 3.8 fold difference between strains) for cytotoxic chemotherapy in NRGS mice (Fig 6E).

## Discussion

We show that RAG-based mice tolerate a busulfan preconditioning regimen in combination with leukemia chemotherapy, expanding the number of samples that can be studied in vivo. RAG-based mice also tolerate multiple cycles of therapy, thereby allowing for more aggressive, realistic modeling. Furthermore, standard AML therapy in RAG mice was 3.8-fold more specific for AML cells, relative to SCID mice, demonstrating an improved therapeutic window for genotoxic agents. We conclude that RAG-based mice represent the new standard for preclinical evaluation of therapeutic strategies involving genotoxic agents.

We did not cure any mice using either our daunorubicin HD or repeated cycle protocols, even in a PDX model of a de novo patient that achieved a MRD(-) remission clinically. This suggests that while we have improved modeling of standard therapy, the models are still not optimized. One difficulty is likely the relative lack of benefit from Ara-C in these models. Ideally, Ara-C would be delivered as a continuous slow infusion by implanted osmotic pumps over the course of a week rather than as several bolus injections. Alternatively, liposomal formulations could also improve efficacy [33]. Supportive care to combat treatment toxicities is also largely absent in PDX models and this may limit the successful implementation of any protocol, particularly if disease burden is high. However, further optimization may not be the preferred approach, since a perfected model would likely make it more difficult to realize a PDX benefit from additional novel therapies.

The ability to tolerate higher chemotherapy doses also suggests that additional cytotoxic agents could be added to the DA backbone. For example, pediatric AML patients are commonly treated with etoposide in addition to DA (so-called ADE induction therapy). The RAG background should allow for expanded chemotherapy modeling in mice. Additionally, targeting anti-apoptotic Bcl-2 familiy proteins along with standard therapy is of great interest, however these inhibitors have been shown to sensitize to anthracyclines [34–36] posing a major challenge for in vivo experiments with SCID mice. The SCID defect would also cause sensitivities to other therapies that induce double strand DNA breaks. As a result, experiments that couple proton therapy to chemotherapy or other sensitizers [37] could be more easily done with RAG-based mice.

The finding of a 3.8-fold improvement of the therapeutic window with RAG relative to SCID mice is similar to the 2–3 fold difference in sensitivity reported between SCID and BALB/c fibroblasts exposed to bleomycin or gamma irradiation, both of which induce double strand DNA breaks [16]. Anthracyclines such as daunorubicin and doxorubicin inhibit the ability of Topoisomerase II to reseal double strand DNA breaks. Given that the SCID mutation renders cells defective in double strand break repair, this likely provides the rationale for increased sensitivity of SCID mice to both the DA and VXPD induction protocols and a worse therapeutic window.

Interestingly, we have found that SCID and RAG mice have similar sensitivities to busulfan conditioning but react differently upon additional genotoxic stress. Busulfan works by induction of intra-strand crosslinks and mono-alkylation of DNA [38], so one might predict repair to be independent of the *Pkrdc*^*scid*^ mutation. However, prior exposure to busulfan further increased the sensitivity of SCID mice to ds-DNA break inducing agents as evidenced by the failure of conditioned NSGS mice to tolerate DA therapy at doses tolerated by naïve NSGS mice. This has clear implications for studies that combine anthracyclines with other DNA damage inducing agents, even if the mechanisms of action are distinct. RAG-based immune-deficient mice should be used for chemotherapy modeling that requires conditioning of mice prior to engraftment.

Although the SCID-associated genotoxic sensitivities are especially severe when ds-DNA break-inducing agents are used, it should be appreciated that some agents that do not damage DNA may increase sensitivity to anthracyclines. For example, the BCL inhibitor venetoclax enhances the effects of ionizing radiation [39]. Experimental MDM2 inhibitors are potent activators of p53 which could be expected to sensitize cells to anthracycline therapy [40, 41]. Furthermore, some compounds may produce unexpected toxicities in the same way, as has been described recently for abemaciclib [42]. These activities are likely to increase non-specific toxicities of standard chemotherapy in SCID-based mouse models, effectively limiting detection and measurement of a pre-clinical therapeutic window.

In the current study we substituted doxorubicin with daunorubicin in order to more closely follow accepted clinical protocols. Surprisingly, we found less efficacy than expected with daunorubicin and a marked improvement of response to doxorubicin over daunorubicin in our PDX models in head to head experiments. This could simply reflect a difference in human and mouse metabolism of the drugs. On the other hand, it could indicate a true difference in the efficacy of these anthracyclines. The optimal dose for individual anthracyclines is different for each drug and there is active study and debate about the relative efficacy between the members of the class. Non-hematopoietic toxicities are an important clinical consideration that must be balanced against the anti-leukemic effect. A retrospective study of childhood cancer survivors demonstrated that daunorubicin resulted in approximately half the risk of future cardiac failure relative to doxorubicin [43]. Doxorubicin has also been found to be associated with more complications due to infections than daunorubicin when given to ALL patients during delayed intensification [44]. Another study with retrospective analysis of a large group of patients found that in children over 3 years of age, doxorubicin was associated with significantly higher rates of induction related mortality, but fewer induction failures than were observed with daunorubicin [45].

Similarly, we have previously used L-asparaginase for PDX ALL induction therapy but switched to pegaspargase in order to update our models to more closely follow practices in pediatric oncology. A large multi-center trial of childhood de novo ALL found that results and toxicities from biweekly pegaspargase were very similar to those observed with weekly intra-muscular injection of native L-asparaginase given after initial induction induced remission [46]. Similarly, a comparison in a relatively low number of adult high-risk ALL patients found no difference in clinical outcomes [47]. In a cohort of relapsed pediatric ALL patients, while pegaspargase demonstrated a prolonged half-life, there was an observed trend towards lower asparagine clearance in the CNS [48]. It remains to be seen whether this substitution has any effects in the PDX setting.

Recently, we have shown that humanized NSGS mice have improved hematopoietic function over humanized NSG mice [49]. Moving forward, it will be important to test immune function in NRGS mice as well, since these mice could be better hosts to build immune therapy models with, particularly if exposure to genotoxic agents is planned. The ability to busulfan condition prior to chemotherapy will be an important advantage for building better models of therapy. For example, conditioning is required for engraftment and humanization with UCB. UCB-transplanted mice might allow for the evaluation of therapies in the context of human immune cells [50].

## Supporting information

S1 Table Summary of PDX models. Age, sex, and stage of disease of the source material used to build each PDX model is listed. The genomic alterations present in the PDX models as well as a description of the latencies in mouse strains with and without busulfan conditioning is also listed.
(XLSX)

## References

[1] Shultz LD, Lyons BL, Burzenski LM, Gott B, Chen X, Chaleff S, et al Human lymphoid and myeloid cell development in NOD/LtSz-scid IL2R gamma null mice engrafted with mobilized human hemopoietic stem cells. Journal of immunology (Baltimore, Md: 1950). 2005;174(10):6477–89. Epub 2005/05/10. 10.4049/jimmunol.174.10.6477 . 15879151

[2] Agliano A, Martin-Padura I, Mancuso P, Marighetti P, Rabascio C, Pruneri G, et al Human acute leukemia cells injected in NOD/LtSz-scid/IL-2Rgamma null mice generate a faster and more efficient disease compared to other NOD/scid-related strains. International journal of cancer. 2008;123(9):2222–7. Epub 2008/08/09. 10.1002/ijc.23772 . 18688847

[3] Wunderlich M, Chou FS, Link KA, Mizukawa B, Perry RL, Carroll M, et al AML xenograft efficiency is significantly improved in NOD/SCID-IL2RG mice constitutively expressing human SCF, GM-CSF and IL-3. Leukemia. 2010;24(10):1785–8. Epub 2010/08/06. 10.1038/leu.2010.158 . 20686503 PMC5439963

[4] Krevvata M, Shan X, Zhou C, Dos Santos C, Habineza Ndikuyeze G, Secreto A, et al Cytokines increase engraftment of human acute myeloid leukemia cells in immunocompromised mice but not engraftment of human myelodysplastic syndrome cells. Haematologica. 2018;103(6):959–71. Epub 2018/03/17. 10.3324/haematol.2017.183202 29545344 PMC6058784

[5] Barve A, Casson L, Krem M, Wunderlich M, Mulloy JC, Beverly LJ. Comparative utility of NRG and NRGS mice for the study of normal hematopoiesis, leukemogenesis, and therapeutic response. Experimental hematology. 2018;67:18–31. Epub 2018/08/21. 10.1016/j.exphem.2018.08.004 30125602 PMC6200600

[6] Wunderlich M, Mizukawa B, Chou FS, Sexton C, Shrestha M, Saunthararajah Y, et al AML cells are differentially sensitive to chemotherapy treatment in a human xenograft model. Blood. 2013;121(12):e90–7. Epub 2013/01/26. 10.1182/blood-2012-10-464677 23349390 PMC3606073

[7] Izumchenko E, Paz K, Ciznadija D, Sloma I, Katz A, Vasquez-Dunddel D, et al Patient-derived xenografts effectively capture responses to oncology therapy in a heterogeneous cohort of patients with solid tumors. Annals of oncology: official journal of the European Society for Medical Oncology. 2017;28(10):2595–605. Epub 2017/09/26. 10.1093/annonc/mdx416 . 28945830 PMC5834154

[8] Zhang CC, Yan Z, Pascual B, Jackson-Fisher A, Huang DS, Zong Q, et al Gemtuzumab Ozogamicin (GO) Inclusion to Induction Chemotherapy Eliminates Leukemic Initiating Cells and Significantly Improves Survival in Mouse Models of Acute Myeloid Leukemia. Neoplasia (New York, NY). 2018;20(1):1–11. Epub 2017/11/25. 10.1016/j.neo.2017.10.008 29172076 PMC5702869

[9] Sperlazza J, Rahmani M, Beckta J, Aust M, Hawkins E, Wang SZ, et al Depletion of the chromatin remodeler CHD4 sensitizes AML blasts to genotoxic agents and reduces tumor formation. Blood. 2015;126(12):1462–72. Epub 2015/08/13. 10.1182/blood-2015-03-631606 26265695 PMC4573869

[10] Sivagnanalingam U, Balys M, Eberhardt A, Wang N, Myers JR, Ashton JM, et al Residual Disease in a Novel Xenograft Model of RUNX1-Mutated, Cytogenetically Normal Acute Myeloid Leukemia. PLoS One. 2015;10(7):e0132375 Epub 2015/07/16. 10.1371/journal.pone.0132375 26177509 PMC4503761

[11] Lee EM, Yee D, Busfield SJ, McManus JF, Cummings N, Vairo G, et al Efficacy of an Fc-modified anti-CD123 antibody (CSL362) combined with chemotherapy in xenograft models of acute myelogenous leukemia in immunodeficient mice. Haematologica. 2015;100(7):914–26. Epub 2015/07/02. 10.3324/haematol.2014.113092 26130514 PMC4486226

[12] Velu CS, Chaubey A, Phelan JD, Horman SR, Wunderlich M, Guzman ML, et al Therapeutic antagonists of microRNAs deplete leukemia-initiating cell activity. The Journal of clinical investigation. 2014;124(1):222–36. Epub 2013/12/18. 10.1172/JCI66005 24334453 PMC3871218

[13] Brana I, Pham NA, Kim L, Sakashita S, Li M, Ng C, et al Novel combinations of PI3K-mTOR inhibitors with dacomitinib or chemotherapy in PTEN-deficient patient-derived tumor xenografts. Oncotarget. 2017;8(49):84659–70. Epub 2017/11/22. 10.18632/oncotarget.19109 29156674 PMC5689564

[14] Bruedigam C, Bagger FO, Heidel FH, Paine Kuhn C, Guignes S, Song A, et al Telomerase inhibition effectively targets mouse and human AML stem cells and delays relapse following chemotherapy. Cell stem cell. 2014;15(6):775–90. Epub 2014/12/07. 10.1016/j.stem.2014.11.010 25479751 PMC4317339

[15] Fulop GM, Phillips RA. The scid mutation in mice causes a general defect in DNA repair. Nature. 1990;347(6292):479–82. Epub 1990/10/04. 10.1038/347479a0 . 2215662

[16] Biedermann KA, Sun JR, Giaccia AJ, Tosto LM, Brown JM. scid mutation in mice confers hypersensitivity to ionizing radiation and a deficiency in DNA double-strand break repair. Proceedings of the National Academy of Sciences of the United States of America. 1991;88(4):1394–7. Epub 1991/02/15. 10.1073/pnas.88.4.1394 1996340 PMC51024

[17] Shultz LD, Lang PA, Christianson SW, Gott B, Lyons B, Umeda S, et al NOD/LtSz-Rag1null mice: an immunodeficient and radioresistant model for engraftment of human hematolymphoid cells, HIV infection, and adoptive transfer of NOD mouse diabetogenic T cells. Journal of immunology (Baltimore, Md: 1950). 2000;164(5):2496–507. Epub 2000/02/29. 10.4049/jimmunol.164.5.2496 . 10679087

[18] Pearson T, Shultz LD, Miller D, King M, Laning J, Fodor W, et al Non-obese diabetic-recombination activating gene-1 (NOD-Rag1 null) interleukin (IL)-2 receptor common gamma chain (IL2r gamma null) null mice: a radioresistant model for human lymphohaematopoietic engraftment. Clinical and experimental immunology. 2008;154(2):270–84. Epub 2008/09/13. 10.1111/j.1365-2249.2008.03753.x 18785974 PMC2612717

[19] Kremer LC, van Dalen EC, Offringa M, Ottenkamp J, Voute PA. Anthracycline-induced clinical heart failure in a cohort of 607 children: long-term follow-up study. Journal of clinical oncology: official journal of the American Society of Clinical Oncology. 2001;19(1):191–6. Epub 2001/01/03. 10.1200/jco.2001.19.1.191 . 11134212

[20] Dohner H, Estey EH, Amadori S, Appelbaum FR, Buchner T, Burnett AK, et al Diagnosis and management of acute myeloid leukemia in adults: recommendations from an international expert panel, on behalf of the European LeukemiaNet. Blood. 2010;115(3):453–74. Epub 2009/11/03. 10.1182/blood-2009-07-235358 . 19880497

[21] Creutzig U, van den Heuvel-Eibrink MM, Gibson B, Dworzak MN, Adachi S, de Bont E, et al Diagnosis and management of acute myeloid leukemia in children and adolescents: recommendations from an international expert panel. Blood. 2012;120(16):3187–205. Epub 2012/08/11. 10.1182/blood-2012-03-362608 . 22879540

[22] Jones L, Richmond J, Evans K, Carol H, Jing D, Kurmasheva RT, et al Bioluminescence Imaging Enhances Analysis of Drug Responses in a Patient-Derived Xenograft Model of Pediatric ALL. Clinical cancer research: an official journal of the American Association for Cancer Research. 2017;23(14):3744–55. Epub 2017/01/26. 10.1158/1078-0432.ccr-16-2392 . 28119366 PMC5943062

[23] Szymanska B, Wilczynska-Kalak U, Kang MH, Liem NL, Carol H, Boehm I, et al Pharmacokinetic modeling of an induction regimen for in vivo combined testing of novel drugs against pediatric acute lymphoblastic leukemia xenografts. PLoS One. 2012;7(3):e33894 Epub 2012/04/06. 10.1371/journal.pone.0033894 22479469 PMC3315513

[24] Benito JM, Godfrey L, Kojima K, Hogdal L, Wunderlich M, Geng H, et al MLL-Rearranged Acute Lymphoblastic Leukemias Activate BCL-2 through H3K79 Methylation and Are Sensitive to the BCL-2-Specific Antagonist ABT-199. Cell reports. 2015;13(12):2715–27. Epub 2015/12/30. 10.1016/j.celrep.2015.12.003 26711339 PMC4700051

[25] Samuels AL, Beesley AH, Yadav BD, Papa RA, Sutton R, Anderson D, et al A pre-clinical model of resistance to induction therapy in pediatric acute lymphoblastic leukemia. Blood cancer journal. 2014;4:e232 Epub 2014/08/02. 10.1038/bcj.2014.52 25083816 PMC4219466

[26] Wunderlich M, Brooks RA, Panchal R, Rhyasen GW, Danet-Desnoyers G, Mulloy JC. OKT3 prevents xenogeneic GVHD and allows reliable xenograft initiation from unfractionated human hematopoietic tissues. Blood. 2014;123(24):e134–44. Epub 2014/04/30. 10.1182/blood-2014-02-556340 24778156 PMC4055932

[27] Wunderlich M, Mulloy JC. Model systems for examining effects of leukemia-associated oncogenes in primary human CD34+ cells via retroviral transduction. Methods in molecular biology (Clifton, NJ). 2009;538:263–85. Epub 2009/03/12. 10.1007/978-1-59745-418-6_13 19277588 PMC2825883

[28] Wei J, Wunderlich M, Fox C, Alvarez S, Cigudosa JC, Wilhelm JS, et al Microenvironment determines lineage fate in a human model of MLL-AF9 leukemia. Cancer cell. 2008;13(6):483–95. Epub 2008/06/10. 10.1016/j.ccr.2008.04.020 18538732 PMC2486365

[29] Lin S, Wei J, Wunderlich M, Chou FS, Mulloy JC. Immortalization of human AE pre-leukemia cells by hTERT allows leukemic transformation. Oncotarget. 2016;7(35):55939–50. Epub 2016/08/11. 10.18632/oncotarget.11093 27509060 PMC5302887

[30] Mulloy JC, Cammenga J, Berguido FJ, Wu K, Zhou P, Comenzo RL, et al Maintaining the self-renewal and differentiation potential of human CD34+ hematopoietic cells using a single genetic element. Blood. 2003;102(13):4369–76. Epub 2003/08/30. 10.1182/blood-2003-05-1762 . 12946995

[31] Townsend EC, Murakami MA, Christodoulou A, Christie AL, Koster J, DeSouza TA, et al The Public Repository of Xenografts Enables Discovery and Randomized Phase II-like Trials in Mice. Cancer cell. 2016;29(4):574–86. Epub 2016/04/14. 10.1016/j.ccell.2016.03.008 27070704 PMC5177991

[32] Hayakawa J, Hsieh MM, Uchida N, Phang O, Tisdale JF. Busulfan produces efficient human cell engraftment in NOD/LtSz-Scid IL2Rgamma(null) mice. Stem cells (Dayton, Ohio). 2009;27(1):175–82. Epub 2008/10/18. 10.1634/stemcells.2008-0583 . 18927475 PMC2696310

[33] Lancet JE, Uy GL, Cortes JE, Newell LF, Lin TL, Ritchie EK, et al CPX-351 (cytarabine and daunorubicin) Liposome for Injection Versus Conventional Cytarabine Plus Daunorubicin in Older Patients With Newly Diagnosed Secondary Acute Myeloid Leukemia. Journal of clinical oncology: official journal of the American Society of Clinical Oncology. 2018;36(26):2684–92. Epub 2018/07/20. 10.1200/jco.2017.77.6112 30024784 PMC6127025

[34] Baranski Z, de Jong Y, Ilkova T, Peterse EF, Cleton-Jansen AM, van de Water B, et al Pharmacological inhibition of Bcl-xL sensitizes osteosarcoma to doxorubicin. Oncotarget. 2015;6(34):36113–25. Epub 2015/09/30. 10.18632/oncotarget.5333 26416351 PMC4742165

[35] Anstee NS, Bilardi RA, Ng AP, Xu Z, Robati M, Vandenberg CJ, et al Impact of elevated anti-apoptotic MCL-1 and BCL-2 on the development and treatment of MLL-AF9 AML in mice. Cell death and differentiation. 2018 Epub 2018/11/25. 10.1038/s41418-018-0209-1 . 30470795 PMC6748137

[36] Teh TC, Nguyen NY, Moujalled DM, Segal D, Pomilio G, Rijal S, et al Enhancing venetoclax activity in acute myeloid leukemia by co-targeting MCL1. Leukemia. 2018;32(2):303–12. Epub 2017/07/29. 10.1038/leu.2017.243 . 28751770

[37] Yu JI, Choi C, Shin SW, Son A, Lee GH, Kim SY, et al Valproic Acid Sensitizes Hepatocellular Carcinoma Cells to Proton Therapy by Suppressing NRF2 Activation. Scientific reports. 2017;7(1):14986 Epub 2017/11/10. 10.1038/s41598-017-15165-3 29118323 PMC5678087

[38] Iwamoto T, Hiraku Y, Oikawa S, Mizutani H, Kojima M, Kawanishi S. DNA intrastrand cross-link at the 5‘-GA-3’ sequence formed by busulfan and its role in the cytotoxic effect. Cancer science. 2004;95(5):454–8. Epub 2004/05/11. 10.1111/j.1349-7006.2004.tb03231.x . 15132775 PMC11158704

[39] O’Steen S, Green DJ, Gopal AK, Orozco JJ, Kenoyer AL, Lin Y, et al Venetoclax Synergizes with Radiotherapy for Treatment of B-cell Lymphomas. Cancer research. 2017;77(14):3885–93. Epub 2017/06/02. 10.1158/0008-5472.CAN-17-0082 28566329 PMC5560265

[40] Miyazaki M, Uoto K, Sugimoto Y, Naito H, Yoshida K, Okayama T, et al Discovery of DS-5272 as a promising candidate: A potent and orally active p53-MDM2 interaction inhibitor. Bioorganic & medicinal chemistry. 2015;23(10):2360–7. Epub 2015/04/18. 10.1016/j.bmc.2015.03.069 . 25882531

[41] Fan Y, Li M, Ma K, Hu Y, Jing J, Shi Y, et al Dual-target MDM2/MDMX inhibitor increases the sensitization of doxorubicin and inhibits migration and invasion abilities of triple-negative breast cancer cells through activation of TAB1/TAK1/p38 MAPK pathway. Cancer biology & therapy. 2018:1–16. Epub 2018/11/22. 10.1080/15384047.2018.1539290 . 30462562 PMC6605999

[42] Naz S, Sowers A, Choudhuri R, Wissler M, Gamson J, Mathias A, et al Abemaciclib, a Selective CDK4/6 Inhibitor, Enhances the Radiosensitivity of Non-Small Cell Lung Cancer In Vitro and In Vivo. Clinical cancer research: an official journal of the American Association for Cancer Research. 2018;24(16):3994–4005. Epub 2018/05/03. 10.1158/1078-0432.Ccr-17-3575 29716919 PMC6137329

[43] Feijen EA, Leisenring WM, Stratton KL, Ness KK, van der Pal HJ, Caron HN, et al Equivalence Ratio for Daunorubicin to Doxorubicin in Relation to Late Heart Failure in Survivors of Childhood Cancer. Journal of clinical oncology: official journal of the American Society of Clinical Oncology. 2015;33(32):3774–80. Epub 2015/08/26. 10.1200/jco.2015.61.5187 26304888 PMC4737860

[44] Schramm F, Zimmermann M, Jorch N, Pekrun A, Borkhardt A, Imschweiler T, et al Daunorubicin during delayed intensification decreases the incidence of infectious complications—a randomized comparison in trial CoALL 08–09. Leukemia & lymphoma. 2019;60(1):60–8. Epub 2018/07/04. 10.1080/10428194.2018.1473575 . 29966458

[45] Buckley JD, Lampkin BC, Nesbit ME, Bernstein ID, Feig SA, Kersey JH, et al Remission induction in children with acute non-lymphocytic leukemia using cytosine arabinoside and doxorubicin or daunorubicin: a report from the Childrens Cancer Study Group. Medical and pediatric oncology. 1989;17(5):382–90. Epub 1989/01/01. 10.1002/mpo.2950170507 . 2677628

[46] Place AE, Stevenson KE, Vrooman LM, Harris MH, Hunt SK, O’Brien JE, et al Intravenous pegylated asparaginase versus intramuscular native Escherichia coli L-asparaginase in newly diagnosed childhood acute lymphoblastic leukaemia (DFCI 05–001): a randomised, open-label phase 3 trial. The Lancet Oncology. 2015;16(16):1677–90. Epub 2015/11/10. 10.1016/S1470-2045(15)00363-0 . 26549586

[47] Ribera JM, Morgades M, Montesinos P, Martino R, Barba P, Soria B, et al Efficacy and safety of native versus pegylated Escherichia coli asparaginase for treatment of adults with high-risk, Philadelphia chromosome-negative acute lymphoblastic leukemia. Leukemia & lymphoma. 2018;59(7):1634–43. Epub 2017/11/23. 10.1080/10428194.2017.1397661 . 29165013

[48] Panetta JC, Gajjar A, Hijiya N, Hak LJ, Cheng C, Liu W, et al Comparison of native E. coli and PEG asparaginase pharmacokinetics and pharmacodynamics in pediatric acute lymphoblastic leukemia. Clinical pharmacology and therapeutics. 2009;86(6):651–8. Epub 2009/09/11. 10.1038/clpt.2009.162 19741605 PMC2831746

[49] Wunderlich M, Chou FS, Sexton C, Presicce P, Chougnet CA, Aliberti J, et al Improved multilineage human hematopoietic reconstitution and function in NSGS mice. PLoS One. 2018;13(12):e0209034 Epub 2018/12/13. 10.1371/journal.pone.0209034 30540841 PMC6291127

[50] Wang M, Yao LC, Cheng M, Cai D, Martinek J, Pan CX, et al Humanized mice in studying efficacy and mechanisms of PD-1-targeted cancer immunotherapy. FASEB journal: official publication of the Federation of American Societies for Experimental Biology. 2018;32(3):1537–49. Epub 2017/11/18. 10.1096/fj.201700740R . 29146734 PMC5892726

